# Controllable synthesis of TiO_2_/graphene composites for human voice recognition in strain sensor

**DOI:** 10.1371/journal.pone.0306866

**Published:** 2024-08-15

**Authors:** Yan Cheng, Ke Wang, Siyi Zhang

**Affiliations:** 1 School of Music and Dance, Xihua University, Chengdu, China; 2 College of Materials Science and Engineering, Sichuan University, Chengdu, China; National Research Centre, EGYPT

## Abstract

Low-dimensional materials have demonstrated strong potential for use in diverse flexible strain sensors for wearable electronic device applications. However, the limited contact area in the sensing layer, caused by the low specific surface area of typical nanomaterials, hinders the pursuit of high-performance strain-sensor applications. Herein, we report an efficient method for synthesizing TiO_2_-based nanocomposite materials by directly using industrial raw materials with ultrahigh specific surface areas that can be used for strain sensors. A kinetic study of the self-seeded thermal hydrolysis sulfate process was conducted for the controllable synthesis of pure TiO_2_ and related TiO_2_/graphene composites. The hydrolysis readily modified the crystal form and morphology of the prepared TiO_2_ nanoparticles, and the prepared composite samples possessed a uniform nanoporous structure. Experiments demonstrated that the TiO_2_/graphene composite can be used in strain sensors with a maximum Gauge factor of 252. In addition, the TiO_2_/graphene composite-based strain sensor showed high stability by continuously operating over 1,000 loading cycles and aging tests over three months. It also shows that the fabricated strain sensors have the potential for human voice recognition by characterizing letters, words, and musical tones.

## 1. Introduction

Human voice recognition technology has emerged as a pivotal innovation with extensive applications in security systems, assistive technologies, and interactive devices. The ability to capture and interpret voice commands accurately and efficiently requires the development of sensitive and reliable sensors. Among these diverse sensors, flexible sensors are designed for integration into wearable devices to enable the detection of human body movements and behaviors. Strain sensors, which boast attributes such as high sensitivity, stretchability, durability, and swift response and recovery times, are particularly noteworthy. These features are pivotal for accurately tracking human motion [1, 2]. Traditional strain sensors, typically crafted from metal foils or semiconductor films, are flexible, stretchable, and exhibit limited elongation capacity [3]. This limitation is attributed to the brittle nature and low specific surface area of the materials used in these sensors, rendering them unsuitable for applications requiring both high sensitivity and a considerable degree of stretchability. In the pursuit of creating strain sensors that meet these demands, researchers have made significant efforts to explore a variety of conductive materials, such as low-dimensional materials and related composites, which are employed as core sensing elements and show promise for overcoming the limitations of traditional sensors [2, 4].

Among the materials explored for enhancing sensor performance, titanium dioxide (TiO_2_) and graphene composites have shown significant promise owing to their unique mechanical and electrical properties. Graphene and related low-dimensional materials [5–8] are promising for strain sensors and other applications [9–12], whereas nanostructured TiO_2_ is known for its stability and sensitivity to strain [13–15]. Combining these two materials, as explored in previous studies [16], has demonstrated enhanced performance in terms of durability, sensitivity, and operational range, making them ideal for dynamic applications such as voice recognition. In recent years, the synthesis of TiO_2_/graphene composites has been approached using various methodologies, including chemical vapor deposition and sol-gel techniques [17–19]. However, these methods often present limitations in terms of scalability, cost, and environmental impact, which sets the stage for exploring a controllable synthesis process that not only addresses these limitations but also tailors the properties of the composites specifically for large-scale, low-cost voice-recognition applications.

In this study, we used an industrial unconcentrated TiOSO_4_ solution to produce TiO_2_ nanoparticles and related TiO_2_/graphene nanocomposites with a high specific surface area, low cost, low energy consumption, and short processing time. The slurries were characterized by hydrolysis yield tests, and the metatitanic acid samples were characterized using Fourier Transform Infrared Microscopy, X-ray diffraction, and the Brunauer-Emmett-Teller method. The calculated hydrolysis time of the first-derivative curve agrees with the experimental turn-gray point time. More importantly, the experimental results suggested that the crystal form and morphology could be readily modified by optimizing the hydrolysis parameters. The prepared TiO_2_/graphene composites exhibited a uniform nanoporous structure and ultrahigh specific area and were suitable for strain-sensor applications. We show that the prepared TiO_2_/graphene nanocomposite-based strain sensors have a high Gauge factor and excellent stability, and can also be used for human voice recognition.

## 2. Materials and methods

### 2.1. Materials

An industrial TiOSO_4_ solution without further concentration was obtained from an industrial factory in Panzhihua, China. The typical composition of the TiOSO_4_ solution was TiO_2_ at 203.94 g L^-1^, F = (effective H_2_SO_4_)/TiO_2_ = 1.91, Ti^3+^ at 1.93 g L^-1^, and Fe/TiO_2_ = 0.39, sable at 450. All other chemical reagents were purchased from Aladdin (Shanghai, China) and used without further purification. A graphene oxide (GO) solution (10 wt%) was purchased from Xianfeng Tech. Ltd., China.

### 2.2. Human participants

Between December 20, 2023, and February 20, 2024, we recruited eight participants, aged 22 to 28 years, to record human voices. This study was conducted according to the guidelines and recommendations of the National Health Commission of the PRC. All experiments involving human participants were approved by the Ethics Committee of Xihua University. Written informed consent was obtained from all participants.

### 2.3. Sample preparation of TiO_2_ and TiO_2_/graphene composites

**Fig 1:**
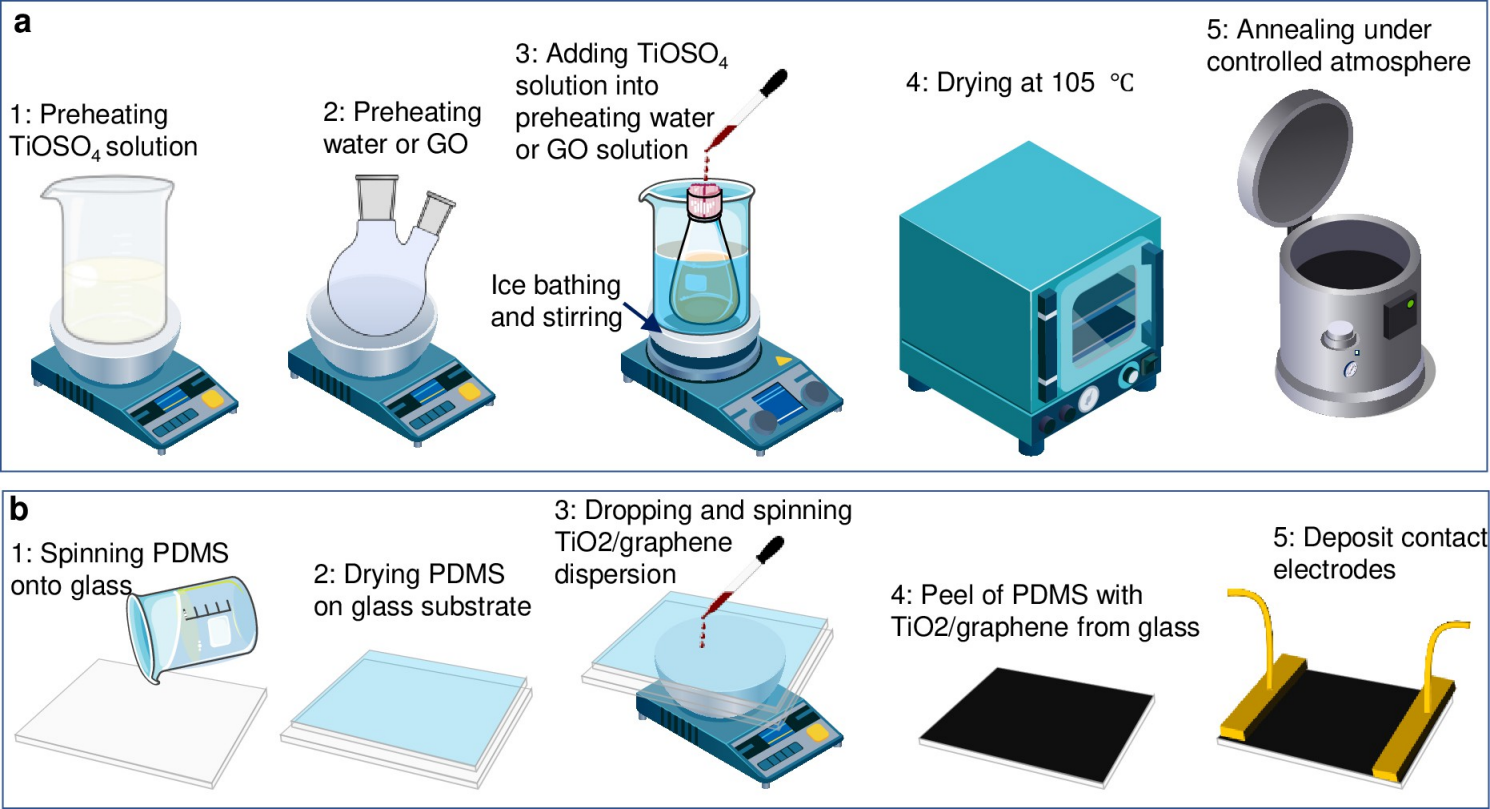
Schematic of experiment setups and workflow employed herein. Schematic key steps of synthesis TiO_2_ and TiO_2_/graphene composites (a), and fabrication process for the TiO_2_/graphene composites-based strain sensor.

The pure TiO_2_ and TiO2/graphene composite samples were prepared using the steps shown in Fig 1A. A volume ratio of 0.2 of pre-added water to the TiOSO_4_ solution was used, with both the TiOSO_4_ solution and the pre-added water individually preheated to temperatures of 96, 98, 100, 102, and 104°C. Subsequently, the preheated TiOSO_4_ solution was slowly added to the pre-added water over a period of approximately 2–3 minutes in a four-necked flask under reflux and stirring, while maintaining the temperature in an oil bath. The slurry was collected when the color of the hydrolysis mixture changed after a few minutes. The slurry was immediately cooled with ice water, followed by the measurement of the Ti^4+^ concentration in the supernatant after centrifugation using a high-speed centrifuge (12,000 rpm, 10 minutes). The sample of metatitanic acid under a hydrolysis temperature of 104°C was dried at 105°C. Finally, the dried sample was annealed at 450°C for 30 minutes to obtain TiO_2_ nanoparticles. The difference in the preparation of the TiO2/graphene composites lies in the addition of a GO solution to replace an identical volume of water in the preparation of the titanium precursor. The mass content of graphene in the prepared composites was 0.8 wt%.

### 2.4. Fabrication of strain sensor

The fabrication of the strain sensor involved the steps shown in Fig 1B. Strain sensors were prepared by coating a 20 mL TiO_2_/graphene composite dispersion onto an elastomeric substrate. The substrate was made from polydimethylsiloxane (PDMS). During the coating process, the PDMS substrate was placed onto a flat oven at 90°C to facilitate the formation of the sensing layer. Finally, silver paste was used to connect the TiO_2_/graphene sensing layer to the copper foil contact electrodes

### 2.5. Characterization and measurements

The sulfate ions, hydroxyl groups, and carbon bonds were confirmed by Fourier Transform Infrared Spectroscopy (FT-IR, Nicolet38, Thermo Electron Corporation, ranging from 4000 to 400 cm^-1^ with a resolution of 2 cm^-1^). The samples were prepared by pressing into thin slices under 20 atm after mixing with pure potassium bromide (KBr), and then detected. Nitrogen adsorption-desorption isotherms of the sample at -196°C were measured using the static volumetric method (3H-2000PS1, Bei Shide, China). The specific surface area and average pore size were estimated using the Brunauer-Emmett-Teller (BET) multipoint and BET methods, respectively. Ti4+ concentration (*C*_0_) in the filtrate was determined using NH_4_Fe(SO_4_)_2_ titration by the Aluminum redox method, and hydrolysis yield (η) was calculated using *η* = (*C*_0_—*C*_t_) / *C*_0_, where *C*_0_ is the initial Ti^4+^ concentration, and *C*_t_ is the Ti^4+^ concentration at t minutes. Particle morphology tests were performed using a Scanning Electron Microscope (SEM; JEOL, JIB 4700F) operated at 20 kV. The as-prepared metatitanic acid was characterized using X-ray diffraction (XRD, model DX-2007) to determine its crystal phase and composition. The diffractometer was operated at 40 kV and a current of 20 mA. Scanning was conducted in the range of 20° to 70° (2*θ*) with a 0.2° step size and an acquisition time of 0.2 seconds per step. The variation in the average size of crystallites (*d*) was determined using Scherrer’s equation: *d* = *λK* / (*βcosθ*), where *λ* represents the wavelength of CuKα radiation, *K* is the particle shape factor, *β* is the full width at half maximum (FWHM) of the intensity peak, and *θ* is the diffraction angle [20]. The electrical performance of the prepared TiO_2_/graphene composites-based strain sensor was monitored using a Keithley 2400 source meter.

## 3. Results and discussion

### 3.1. Direct synthesis of pure TiO_2_ and TiO_2_/graphene composite using industrial raw materials

**Fig 2:**
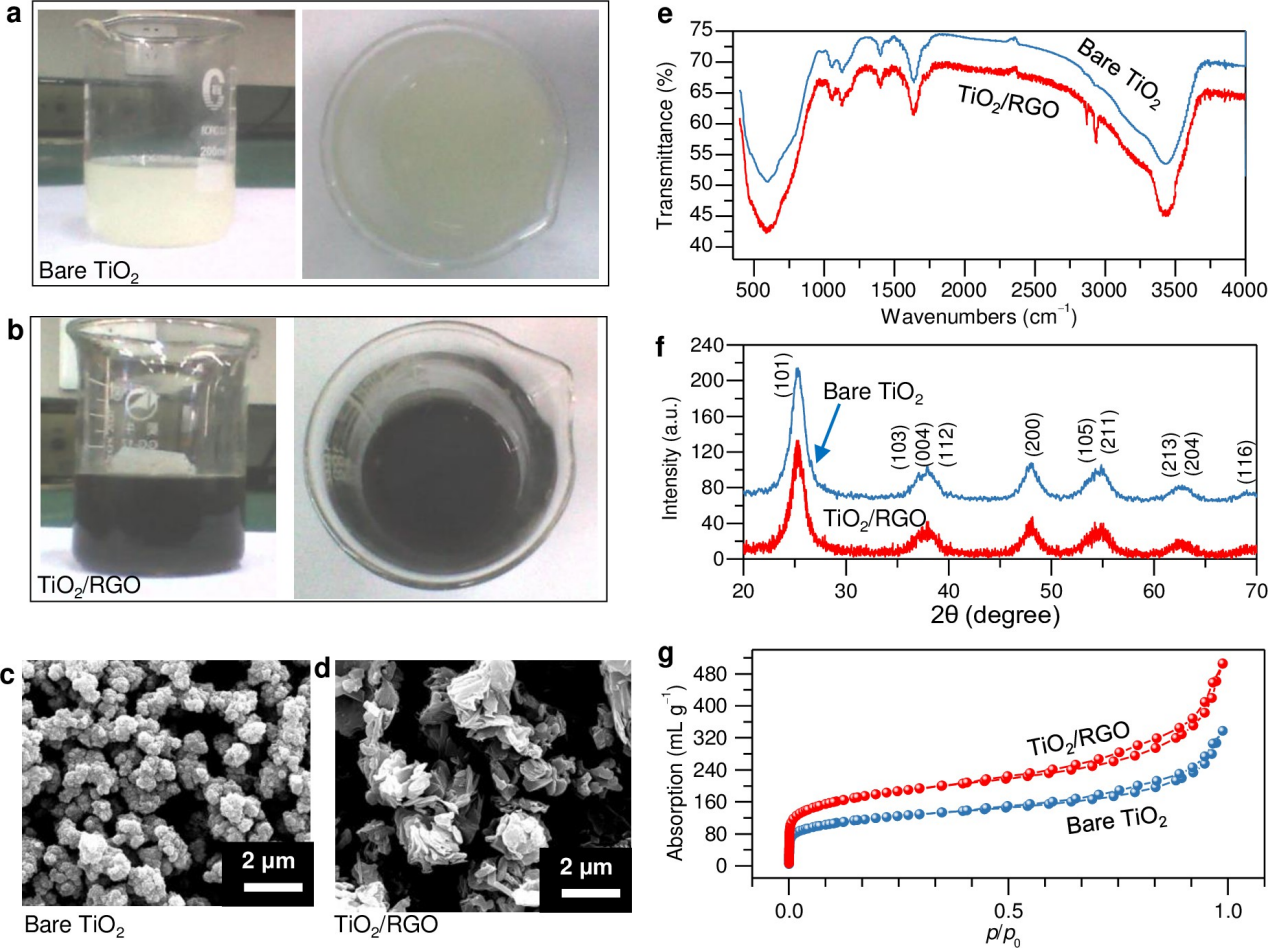
Comparison of prepared pure TiO_2_ nanoparticles and TiO_2_/graphene composites. (**a, b**) Optical images of prepared sample dispersion in ethanol absolute. (**c, d**) SEM images. FT-IR curves (**e**), XRD plots (**f**) and BET tests (**g**) for prepared samples.

Fig 2A and 2B initially show the optical images of prepared TiO_2_ nanoparticles and TiO_2_/graphene composites, both exhibited within ethanol dispersions. More quantitative information about the sample preparation comparison between pure TiO_2_ and TiO_2_/graphene composites can be found by examining their microstructures. Fig 2C and 2D clearly display the SEM images for the above two types of samples, indicating that the reduced GO sheets are wrapped with TiO_2_ nanoparticles. The FT-IR spectra are shown in Fig 2E. Compared with the peak shift of SO_4_^2-^ ions stretching vibration absorption at 1100 cm^-1^, the peak shifts of S = O stretching vibration absorption at 1057.1 cm^-1^ and 1128.2 cm^-1^ show that SO_4_^2-^ ions form stable chemical bonds in metatitanic acid rather than being completely present in the H_2_SO_4_ format. The peak at 3423.7 cm^-1^ represents the vibration absorption of hydroxyl groups, indicating that hydrogen bonds exist in metatitanic acid molecules. This peak around 3423 cm^-1^ may stem from the active centers of O_2_- radicals formed on the surface of anatase crystals during the hydrolysis process, coupled with SO_4_^2-^ ions in a bidentate ligand on the surface of metatitanic acid particles. After hydrothermal processing, we also note significant differences from pure TiO_2_ in our prepared composites consisting of TiO_2_ nanoparticles and reduced graphene oxides (RGO). Compared with pure TiO_2_, the FT-IR spectra of the composite exhibit two distinct peaks at around 1400 and 3400 cm^-1^, which are mainly derived from the C = C bonds. Additionally, a weak peak around 1726 cm^-1^ also appears in the TiO_2_/graphene composite FT-IR spectroscopy, mainly due to the O-H bond in RGO.

Fig 2F shows the diffraction peaks of the obtained TiO_2_ particles with and without RGO, indicating a high degree of crystallization in the anatase phase (ICSD No. 009855). The diffraction peak at approximately 23.76° can be attributed to the (0 0 2) phase of reduced graphene oxide, which is relatively weak mainly because RGO sheets are wrapped within TiO_2_ nanoparticles [18, 21]. The average crystal size of the TiO_2_ nanoparticles was estimated to be approximately 20 nm, derived from the diffraction peak at approximately 25.3° on the (1 0 1) plane using the Scherrer equation [22–24]. This finding agrees well with the morphological characterization presented in the SEM image (Fig 2D). However, the XRD characteristics of graphene are not clear in the spectra, mainly because of the screening effect of unique structures; that is, graphene is wrapped by TiO_2_ nanoparticles, and the components of graphene can be confirmed by the SEM image and FT-IR characterization mentioned above.

We further analyzed the pore distribution of metatitanic acid using nitrogen adsorption-desorption isotherms. As shown in Fig 2G, narrow and long hysteresis loops are clearly observed in the samples, indicating porous structural compounds with a wide pore size distribution. The specific surface area of the prepared pure TiO_2_ samples is approximately 360 m^2^ g^-1^, and the average pore size is approximately 5.2 nm. Moreover, we can attribute the long channels and narrow hysteresis loop to the strong crosslinking of particles and the high content of H_2_O and SO_4_^2-^ in metatitanic acid. After the addition of RGO, the specific surface area of prepared composites significantly increases to approximately 486 m^2^ g^-1^ due to the ultrahigh surface to volume ratio of RGO.

### 3.2. Controllable synthesis of TiO_2_ nanoparticles

The hydrolysis of the industrial TiOSO_4_ solution is a complex physicochemical process [25] that determines the product structure and ultimately affects the performance and application of the titanium dioxide pigment. Most studies on the kinetics and mechanisms aim to understand the hydrolysis process and control the aggregate size of metatitanic acid, which is rarely related to the controllable synthesis of TiO_2_ particles.

**Fig 3:**
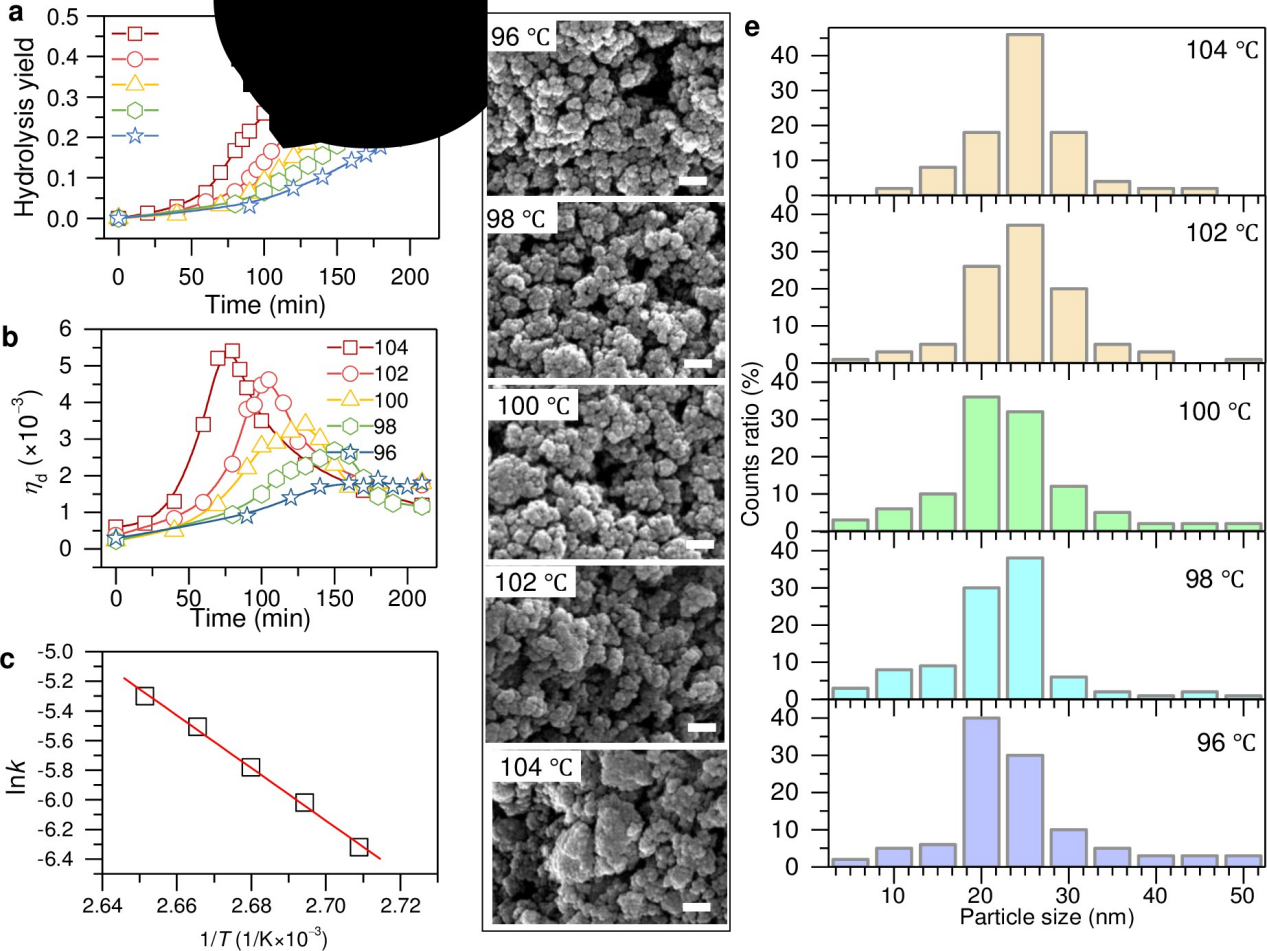
Controllable synthesis of TiO_2_ nanoparticles by tuning the hydrolysis temperature from 96 to 104°C. (**a**) Hydrolysis yield and (**b**) related derivative values as function of reaction time with different thermal temperatures. (**c**) Calculated hydrolysis velocity constant as function of hydrolysis temperature. (**d**) SEM images. (**e**) Particle size distribution.

To gain insights into the controlled synthesis of TiO_2_ nanoparticles, Fig 3A depicts the hydrolysis yield curves characterized by a typical "S" shape, indicating that the hydrolysis yield decreases as the hydrolysis temperature is lowered, and conversely, it gradually increases with rising hydrolysis temperature [26]. Hydrolysis occurs in three distinct phases: induction, active hydrolysis, and maturation. Initially, in the induction phase, there is high activity of the primary crystal nuclei, yet hydrolysis progresses slowly owing to the limited number of nuclei present. As the process enters the active hydrolysis phase, the rate of hydrolysis increases, following substantial nucleation in the induction phase. This phase is dominated by the growth of particle surfaces and a direct correlation between the hydrolysis yield and time, rendering the reaction first-order. Surface diffusion is the controlling factor during the maturation phase. The dwindling concentration of Ti^4+^ ions, increasing acidity, decreasing boiling points, and reducing supersaturation levels collectively slow the hydrolysis rate. Notably, an increase in the hydrolysis temperature shortens the induction and active hydrolysis phases, while extending the maturation phase, thereby markedly enhancing the hydrolysis rate.

The derivative curves of the hydrolysis yield over time were derived from the time-dependent hydrolysis yield curve, as illustrated in Fig 3B. The turn-gray point is the fastest time of change in the hydrolysis rate, which is the maximum of the derivative of the hydrolysis rate. Therefore, hydrolysis is slow during the induction period but forms a large number of secondary nuclei with higher activity. The hydrolysis rate then increases rapidly and reaches a maximum. Once the aggregation between the metallic particles increases, the active sites rapidly decrease on the surface of the particles after turning the gray point, thereby slowing down the hydrolysis rate.

Fig 3C shows the calculated hydrolysis rate based on the Arrhenius equation [27, 28]. With fitting at different temperatures (*T*), the hydrolysis rate constant *k* follows the relations of $\ln k=41.8-1.8\times 10^{4}\frac{1}{T}$, where the goodness of fit *R*^2^ = 0.997. Thus, we can further calculate that the pre-exponential factor *k*_0_ is on the order of 10^18^ min^−1^ and the activation energy *E*_a_ isapproximately 150 kJ mol^−1^, respectively. This ultrafast hydrolysis rate and relatively low activation energy have reached the comparable level of the latest experiment [26]. The data show that the hydrolysis rate of TiOSO_4_ solution is precisely dominated by the hydrolysis temperature.

Next, we focus on the morphology of the prepared TiO_2_ nanoparticles after annealing at 450°C. This clearly shows that the morphology of the obtained TiO_2_ nanoparticles can be readily controlled by hydrolysis, as shown in Fig 3D. The average particle size ranged from approximately 20 to 30 nm when the hydrolysis temperature was changed, as depicted in Fig 3E. This sample route offers great potential for large-scale production of controllable TiO_2_ nanoparticles at a low cost.

### 3.3. Performance characterization of TiO_2_/graphene composite-based strain sensor

In recent years, the development and enhancement of strain sensors have received significant attention in the fields of materials science and engineering, largely due to their extensive applications in wearable electronics, structural health monitoring, human motion detection, and smart textiles. Among the plethora of materials explored for this purpose, composite materials, particularly those based on titanium dioxide and reduced graphene oxide, have emerged as promising candidates due to their unique properties and synergistic effects. This exploration led to a rigorous evaluation of strain sensors made from TiO_2_/RGO composite materials, the results of which underscore the remarkable potential of these materials for fabricating high-performance, reliable sensors.

These strain sensors were meticulously assessed using a sophisticated motorized controller, the WNMC-S 800 Motion Controller, to apply various strains to the sensors. The electrical properties of the fabricated strain sensors were measured in ambient air at room temperature. This process involves numerous cycles of stretching and releasing, thereby subjecting the sensors to different degrees of strain. A pivotal aspect of this evaluation was the measurement of the resistance changes in the sensors (Δ*R*/*R*_0_), where *R*_0_ represents the initial resistance of the sensor, and Δ*R* denotes the change in resistance due to applied strain. This approach enabled a detailed analysis of how the resistance of the sensors varied with the strain, which is critical for understanding their sensitivity and performance.

**Fig 4:**
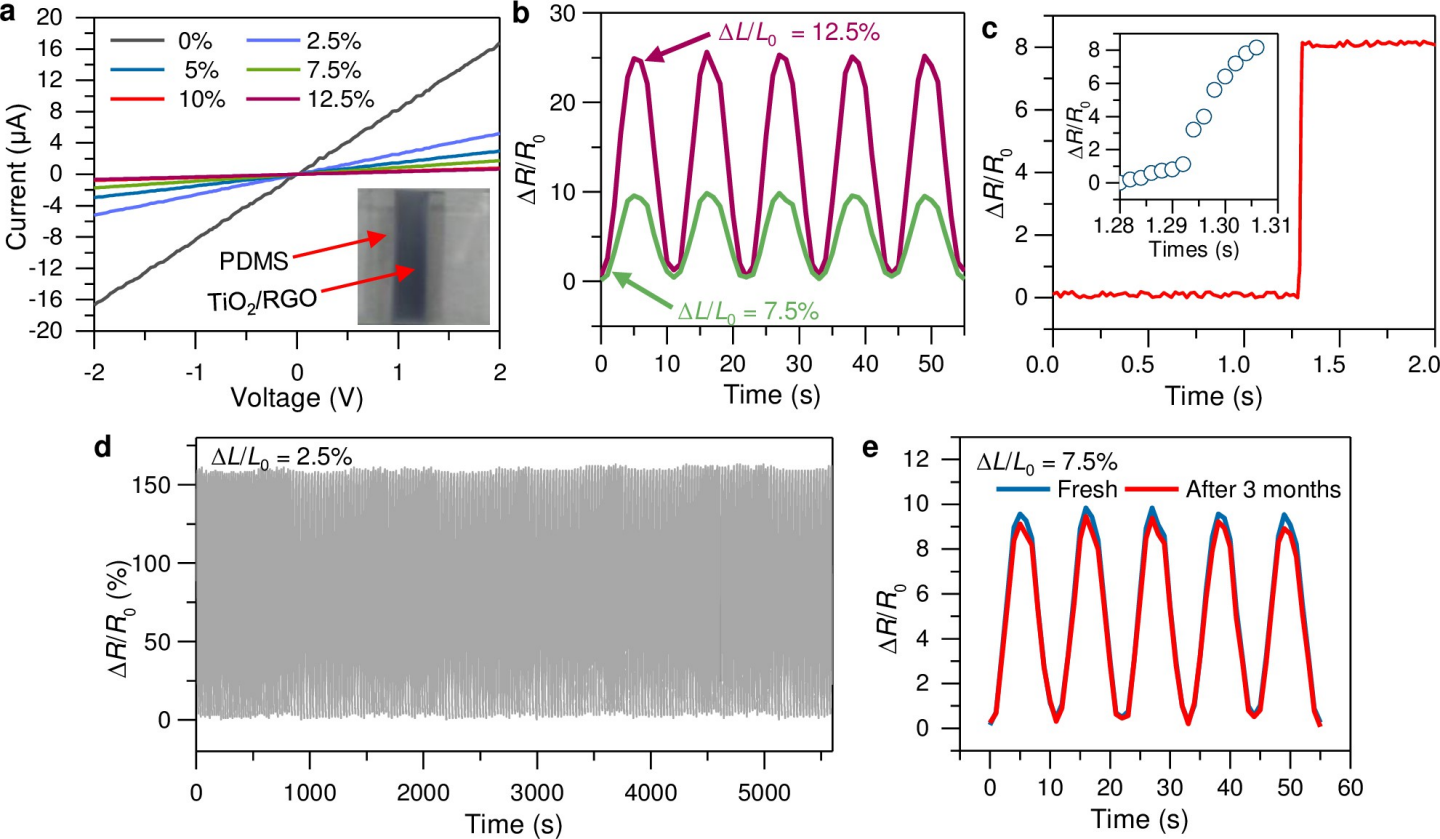
Piezoresistive performance of stain sensors using TiO_2_/RGO composites. (**a**) Demonstration of the *I*-*V* characteristics at varying levels of strain applied to the TiO_2_/RGO composite strain sensor. (b) Resistance changes in the sensors at 7.5% and 10% strain. (c) Resistance change after a sudden increase in pressure input. The inset is the enlarged view of the sudden change in resistance depending on input pressure. (d) Resistance behavior across 1,000 cycles, surpassing 5,000 seconds of loading and unloading at a 2.5% strain. (e) Comparison of the resistance response of the newly created strain sensor in its original condition and after a 3-month period.

One of the key findings of this investigation, as illustrated in Fig 4A, was the establishment of current-voltage *(I-V*) profiles for varying strain intensities. These profiles revealed a consistent level of resistance to each strain. However, under strain, the gradient of the *I-V* curve diminished, indicating an increase in resistance. This phenomenon is crucial because it demonstrates the capability of the sensors to detect and measure strain through changes in electrical resistance, which is a fundamental requirement for strain sensors.

Further analysis was conducted to evaluate the performance of the sensors at various strain magnitudes, focusing on resistance variations to gauge the reliability of the sensor output. The results presented in Fig 4B highlight the remarkable stability of the output signals across all measurements, with no significant deviations detected. This stability is paramount for strain sensors because it ensures consistent and reliable readings over time.

An important metric for assessing the performance of strain sensors is the Gauge factor (GF), which quantifies the sensitivity of a sensor to strain. It is calculated using the formula GF = (Δ*R*/*R*_0_)/(Δ*L*/*L*_0_), where Δ*L* represents the change in the length of the sensor, and *L*_0_ is the initial length. In this study, GF values of 129 and 252 were recorded at 7.5% and 10% strains, respectively, with GF values ranging between 116 and 197. These values are particularly noteworthy as they significantly surpass the performance metrics of previously documented stretchable sensors, which reported GF ranges from 0.5 to 90, and even some graphene-based strain sensors, with GF values between 10 and 197. This demonstrates the exceptional sensitivity of the TiO_2_/RGO composite-based sensors to strain, positioning them as superior alternatives to many existing sensor materials. The unique piezoresistive characteristics of the TiO_2_/RGO composite-based elastic conductors can be attributed to structure-dependent contact mechanisms. Similar behavior has been observed in other low-dimensional materials, such as graphene-carbon nanotube hybrids [3] and graphene-nanocellulose nanopapers [29]. Moreover, the developed sensor offers the advantage of a rapid response to applied pressure, as shown in Fig 4C. The response time was measured to be approximately 0.031 s when subjected to a sudden pressure input. Notably, this response time is not the highest but is comparable to those reported for other sensors [30, 31].

In addition to their sensitivity, the TiO_2_/RGO composite-material-based sensors demonstrated exceptional long-term stability and enhanced durability. Fig 4D shows the consistent response over 1,000 stretching cycles at 2.5% strain, exceeding 5,000 seconds of testing. This level of stability indicates the robustness of the sensor and its ability to maintain its performance over extensive use. Moreover, an examination after a 3-month exposure to ambient conditions, as illustrated in Fig 4E, showed only a sub-5% decline in the current compared to the initial measurements. This finding underscores the superior stability and reliability of the developed strain sensors, even under long-term environmental exposure.

Advancements in strain sensors based on TiO_2_/RGO composite materials represent a significant leap forward in the field of sensor technology. Their ability to combine high sensitivity, remarkable stability, and excellent durability makes them ideal for a wide range of applications, from wearable technology to infrastructure monitoring. The meticulous testing and positive outcomes highlighted in this study pave the way for further research and development, with the potential to revolutionize the design and implementation of strain sensors in various sectors.

### 3.4. Human voice recognition of TiO_2_/graphene composite-based strain sensor

The development and implementation of advanced strain sensors, particularly those fabricated from TiO_2_/RGO composites, represent a significant leap forward in the field of wearable technology and human-machine interaction. These sensors, characterized by their exceptional piezoresistive performance, offer an innovative solution for detecting and monitoring human movement through subtle changes that occur within the structure of the skin owing to deformations, such as stretching and bending. For instance, skin deformation leads to alterations in the microcracks and micropores within the film of the sensor, enabling it to act as a highly responsive, skin-compatible strain sensor. This capability is pivotal for applications that require precise monitoring of human movements, including vocal expressions. The innovative use of TiO_2_/RGO composites in these sensors sets them apart from traditional strain sensors, which are typically based on metal foils or silicon. Composite-based sensors excel in several critical aspects, including stretchability and durability, making them superior for long-term dynamic applications.

**Fig 5:**
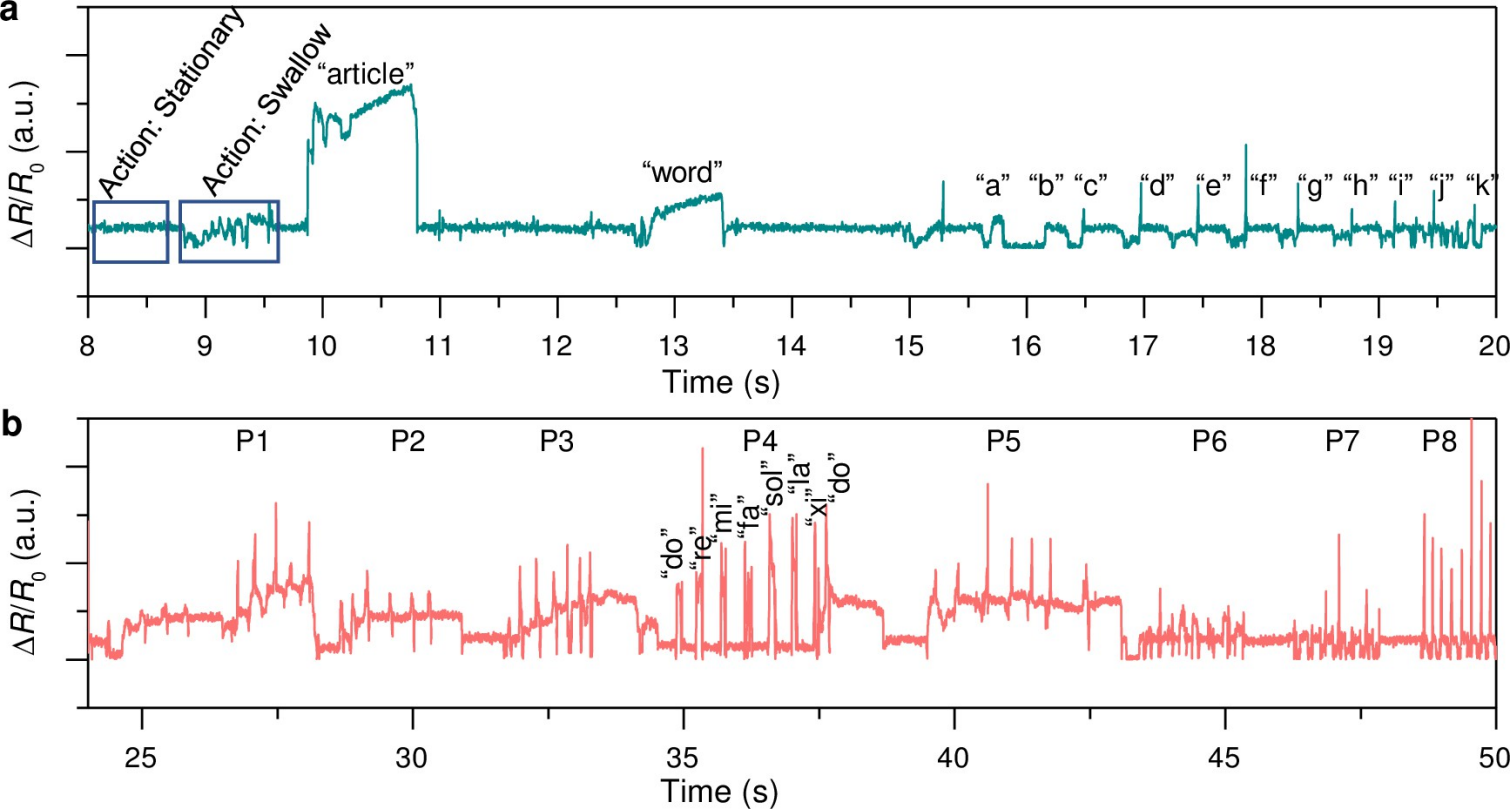
Human voice application of stain sensors using TiO_2_/RGO composites. (**a**) Letters and words recognition of a single person. (**b**) Music tones recognition of eight persons.

The high sensitivity of TiO_2_/RGO composite-based strain sensors, coupled with their straightforward manufacturing process, renders them highly suitable for a broad range of applications, particularly in fields that benefit from human-machine interfaces and advanced voice-monitoring systems. These applications range from assistive technology for individuals with speech impairments to advanced control systems in robotics and virtual reality environments where nuanced human input is necessary for effective interactions. To demonstrate the practical applications of these advanced strain sensors, a specific implementation was demonstrated by attaching a TiO_2_/RGO composite-based strain sensor to the front of the neck to monitor speech. This demonstration, as illustrated in Fig 5A, revealed the ability of the sensor to distinctly capture the pronunciation of the alphabet from "a" to "k," as well as words such as "article" and "word," and even complex actions like swallowing. This level of detail and sensitivity highlights the potential of the sensor for voice recognition and monitoring applications, offering new avenues for both medical diagnostics and interactive technologies.

Furthermore, these sensors have shown capability for complex voice-monitoring tasks. They effectively detected subtle skin movements along the neck and translated them into consistent and reliable resistance signals. This feature is demonstrated in Fig 5B, which shows the acute sensitivity and distinctive current responses of the sensor during the vocalization of musical tones by eight different individuals. This experiment underscores the exceptional performance of the strain sensors in recognizing and differentiating various vocal tones and sounds, further illustrating their potential for use in advanced voice-recognition systems. The superior performance of the TiO_2_/RGO composite-based strain sensors in detecting and monitoring human voices and movements opens new possibilities for several cutting-edge applications. By improving the quality of life for individuals with disabilities and enhancing the interface between humans and machines, these sensors can pave the way for more natural and intuitive interactions. Their high sensitivity, durability, and flexibility, combined with their ease of fabrication, make them a promising solution for the next generation of wearable technology and human-machine interfaces. Thus, the development of TiO_2_/RGO composite-based strain sensors has marked a significant advancement in the fields of wearable sensors and human-machine interaction technology. With their unparalleled sensitivity and ability to monitor complex human movements and vocal expressions, these sensors are ready to revolutionize how we interact with technology and each other, making communication and control more seamless and intuitive than ever before.

## 4. Conclusions

In summary, we have controllably prepared TiO_2_ nanoparticles and related TiO_2_/graphene composites directly using an industrial TiOSO_4_ solution via a hydrothermal method. The particle and core sizes of the TiO_2_ nanoparticles could be controlled by changing the hydrolysis temperature, allowing for the preparation of TiO_2_ nanoparticles with a uniform size distribution and consistent shape. The hydrolysis kinetics and mechanisms were investigated by hydrolyzing an unconcentrated industrial TiOSO_4_ solution. We demonstrate that the prepared TiO_2_/graphene composites can be used for strain-sensor applications with a high GF of up to 252 and excellent stability, continuously operating over 1,000 cycles exceeding 5,000 seconds and aging for over three months. We also show that the fabricated TiO_2_/graphene composite-based strain sensors recognize human voices, including letters, words, and even complex music tones. These observations potentially provide a promising route for the high-performance photocatalytic preparation of TiO_2_-based nanostructured composites at large scale and low cost, and pave the way for the development of smart electronic devices. However, this research mainly focused on the controllable synthesis of TiO_2_/graphene composites for strain-sensor applications, and some proposals should be considered to further enhance the scope and applicability of this technology, including optimizing sensing materials, investigating durability and longevity, and integrating with other technologies. By addressing these challenging areas, future research can build on the foundation of the simple fabrication of strain sensors demonstrated herein, pushing the boundaries of what is possible with TiO_2_/graphene composites in voice recognition and beyond.
