# Correction: Controllable synthesis of TiO_2_/graphene composites for human voice recognition in strain sensor

**DOI:** 10.1371/journal.pone.0320024

**Published:** 2025-03-05

**Authors:** Yan Cheng, Ke Wang, Siyi Zhang

Fig 3 is uploaded incorrectly. Please see the correct Fig 3 here.

**Fig 3:**
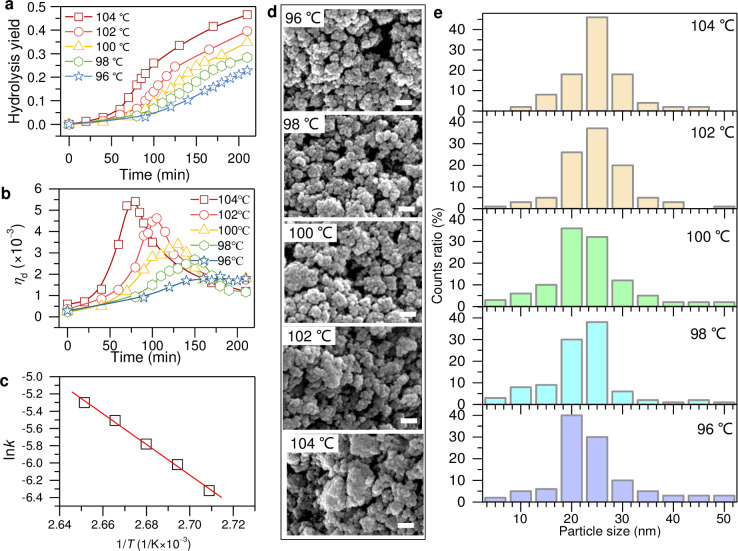
Controllable synthesis of TiO_2_ nanoparticles by tuning the hydrolysis temperature from 96 to 104°C. (a) Hydrolysis yield and (b) related derivative values as function of reaction time with different thermal temperatures. (c) Calculated hydrolysis velocity constant as function of hydrolysis temperature. (d) SEM images. (e) Particle size distribution.
